# Neural correlates of vocal initiation in the VTA/SNc of juvenile male zebra finches

**DOI:** 10.1038/s41598-021-01955-3

**Published:** 2021-11-17

**Authors:** Shin Yanagihara, Maki Ikebuchi, Chihiro Mori, Ryosuke O. Tachibana, Kazuo Okanoya

**Affiliations:** 1 Graduate School of Arts and Sciences, The University of Tokyo, Tokyo, Japan; 2 RIKEN Center for Brain Science, Saitama, Japan; 3 Department of Molecular Biology, Faculty of Pharmaceutical Sciences, Teikyo University, Tokyo, Japan; 4 Present Address: Advanced Comprehensive Research Organization, Teikyo University, Tokyo, Japan

**Keywords:** Basal ganglia, Learning and memory

## Abstract

Initiation and execution of complex learned vocalizations such as human speech and birdsong depend on multiple brain circuits. In songbirds, neurons in the motor cortices and basal ganglia circuitry exhibit preparatory activity before initiation of song, and that activity is thought to play an important role in successful song performance. However, it remains unknown where a start signal for song is represented in the brain and how such a signal would lead to appropriate vocal initiation. To test whether neurons in the midbrain ventral tegmental area (VTA) and substantia nigra pars compacta (SNc) show activity related to song initiation, we carried out extracellular recordings of VTA/SNc single units in singing juvenile male zebra finches. We found that a subset of VTA/SNc units exhibit phasic activity precisely time-locked to the onset of the song bout, and that the activity occurred specifically at the beginning of song. These findings suggest that phasic activity in the VTA/SNc represents a start signal that triggers song vocalization.

## Introduction

Dopamine neurons in the midbrain ventral tegmental area (VTA) and substantia nigra pars compacta (SNc) are involved in a variety of functions, including reward prediction error coding, motivation, arousal regulation, and voluntary movement^1–5^. Through several studies, accumulated neurophysiological data has shown that midbrain dopaminergic neurons encode reward prediction error signals important for reinforcement learning^3,6–8^. Moreover, recent studies in rodents have shown that midbrain dopaminergic neurons exhibit movement-related activity^9–12^. More specifically, the phasic activity of dopaminergic neurons associated with movement initiation has recently received much attention^4^. Thus, the function of dopamine in behavior is still not fully elucidated. Midbrain dopaminergic neurons show a transient increase or decrease in activity at the initiation of voluntary movements such as sequential lever-pressing or locomotion, and that activation of dopaminergic neurons promotes movement initiation^9–11,13,14^. These studies highlight the critical role of the midbrain dopaminergic system in a range of movements, particularly initiation of self-paced movement. On the other hand, although dopaminergic regulation of vocalization has been extensively studied in songbirds^15–17^, it is still unclear whether the midbrain dopaminergic system is involved in the initiation of learned vocalizations.

Songbirds are a well-suited animal species to study the neural mechanisms underlying the initiation and execution of learned vocalizations. Previous studies of the song system nuclei have demonstrated preparatory activity for singing in the premotor nucleus HVC^18–21^ as well as in Area X and LMAN, which are part of the cortico-basal ganglia circuitry^22–25^. While neural activity of these brain areas is important for the preparation and execution of song, it is unclear where in the brain a start signal for song vocalization might come from. Two brain areas that could potentially provide a start signal for song are the midbrain VTA and SNc. Neurons in the VTA/SNc send axons to the song system nuclei^26–29^, and the song system nuclei have an abundance of catecholaminergic terminals and dopamine receptors^30,31^. Moreover, basal ganglia-projecting VTA dopaminergic neurons encode performance errors^32^ and manipulations of this dopaminergic pathway have been shown to affect vocal learning^33–35^. These studies underscore the importance of the VTA dopaminergic system in vocal learning in songbirds; nevertheless, the role of the dopaminergic system in the initiation of song vocalization has not been verified. Juvenile male zebra finches practicing their songs vocalize freely without external stimuli, such as female birds^36^. Thus, by using juveniles in our study, we can test whether the VTA/SNc is involved in the initiation of self-paced vocalizations independent of social context. Here we examined whether VTA/SNc neurons exhibit activity related to the initiation of song by recording extracellular single unit activity from singing juvenile zebra finches.

## Results

**Figure 1:**
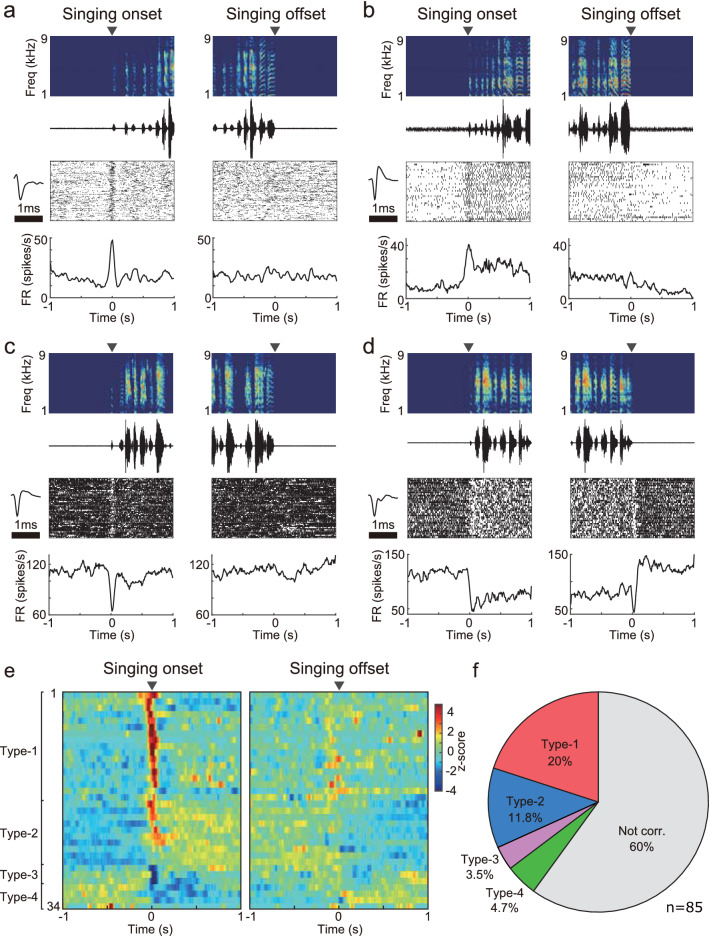
Singing-related activity in VTA/SNc from juvenile zebra finches. (**a**) Example of a type-1 unit during singing. Spike raster plot and PETH aligned to the song bout onset (left) and offset (right). Spectrograms and amplitude oscillograms of song are shown on the top. Inset denotes mean spike waveform. Note the increased burst firing only at the onset of singing. (**b**) Example of a type-2 unit. (**c**) Example of a type-3 unit. Note the brief pause in firing only at the onset of singing. (**d**) Example of a type-4 unit. (**e**) Summary of singing-related VTA/SNc units. The firing rate of each neuron was normalized to a z-score, and aligned to the singing onset (left) and offset (right). Each row represents data from an individual VTA/SNc unit. (**f**) Proportion of singing-related units in VTA/SNc.

**Figure 2:**
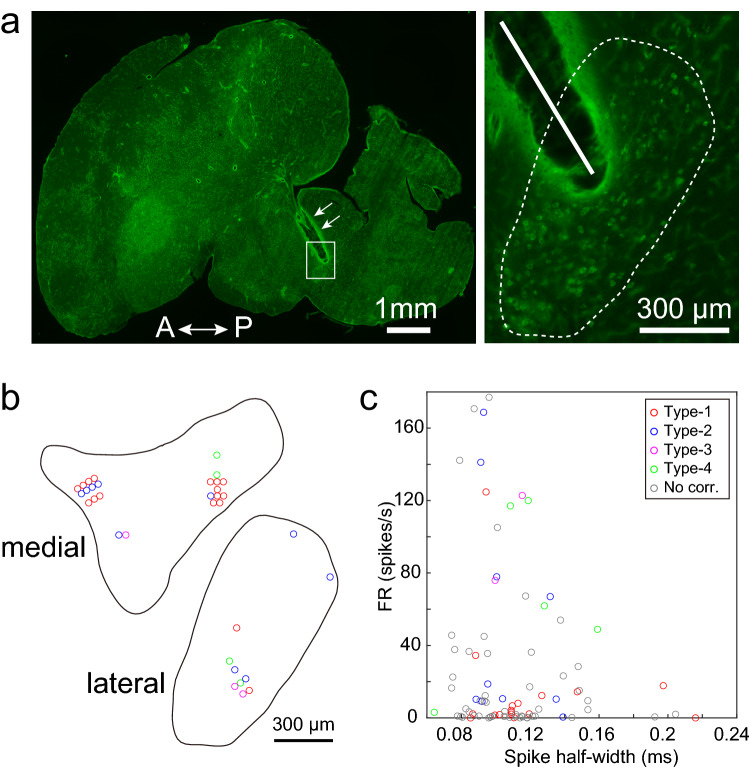
Histological and electrophysiological confirmation of VTA/SNc single units. (**a**, **b**) Histological verification of the recording sites. (**a**) left, Low magnification of a zebra finch sagittal brain section stained with an antibody against tyrosine hydroxylase (TH). A: anterior, P: posterior. Arrows indicate an electrode track. Right, Magnified view of the area enclosed by the white square in the left panel. A white line indicates an electrode track that entered the TH-positive cell population. The TH-positive cell population is surrounded by a white dotted line which corresponds to the ‘lateral’ brain section in (**b**). (**b**) Recording sites from all birds (n = 6) are shown in the example sagittal sections. Each color corresponds to the neuron types (Red: Type-1, Blue: Type-2, Magenta: Type-3, Green: Type-4). Recording sites where singing-related units were obtained are shown. (**c**) Relationship between spontaneous firing rate and spike half-width. Each circle represents data from a single neuron. Red: Type-1, Blue: Type-2, Magenta: Type-3, Green: Type-4, Gray: No correlation.

We recorded the neural activity in VTA/SNc from juvenile male zebra finches while birds were singing spontaneously (n = 85 single units, 6 birds). In this study, we focused on the analysis of neural activity before and during singing. Peri-event time histograms (PETH) and z-scored firing rates were calculated based on the alignment to the onset or offset of the song bout (Fig. 1). We observed that VTA/SNc single units exhibited changes in activity while a bird was singing (34/85 single units, 6 birds, Friedman test, p < 0.05), and these single units were classified into four types based on the activity profiles. Seventeen single units were classified as type-1, which showed a phasic increase in firing rate at the onset of the song bout, followed by a rapid decline in firing rate during singing (Fig. 1a,e,f, type-1, 4 birds). Ten single units were classified as type-2, which showed a phasic increase in firing rate at the onset of the song bout, and the firing rate was sustained throughout the singing period (Fig. 1b,e,f, type-2, 5 birds). On the other hand, other VTA/SNc single units showed a decrease in firing rate during singing. Three single units were classified as type-3, which exhibited a brief pause of activity at the onset of the song bout (Fig. 1c,e,f, type-3, 2 birds). Four single units were classified as type-4, which showed a decrease in firing rate throughout the singing period (Fig. 1d,e,f, type-4, 2 birds). These single units were recorded within the VTA/SNc complex, which has a dense population of TH-positive cells (Fig. 2). Based on the analysis of spontaneous firing rate and spike width, we confirmed that VTA/SNc contains both broad spike and narrow spike units, which is consistent with previous studies in zebra finch VTA/SNc^29,34,37,38^. Broad spike units tended to show low spontaneous firing rates in vivo (< 10–15 Hz)^34,38^, thus indicating dopaminergic neurons (Fig. 2c). On the other hand, narrow spike units tended to show variable firing rates, indicating non-dopaminergic neurons.

**Figure 3:**
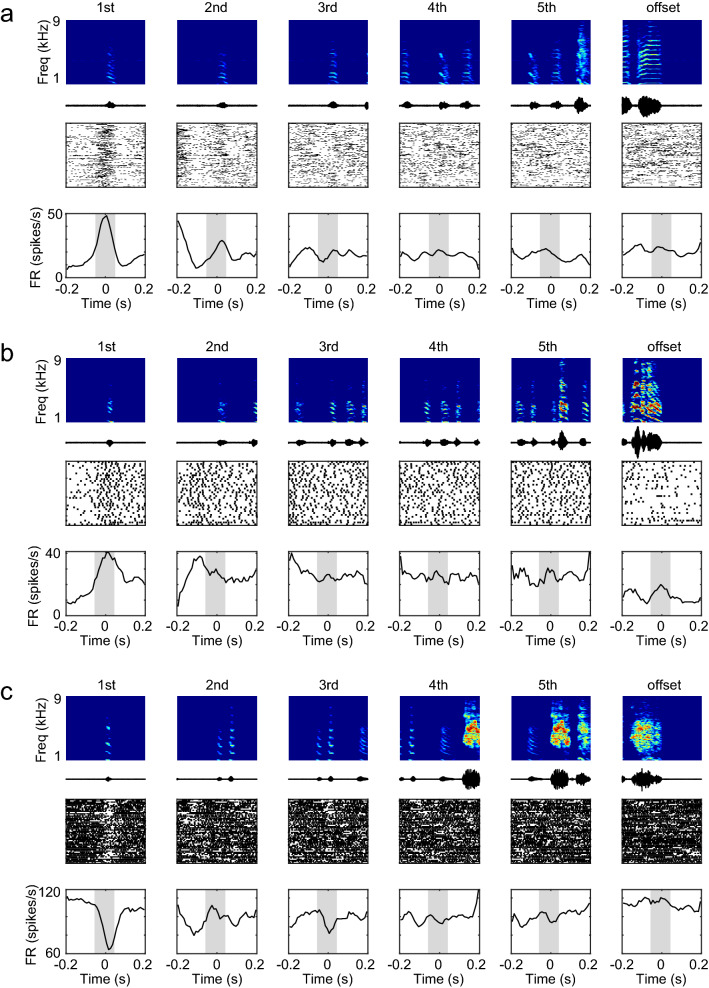
Type-1 and type-3 neurons exhibited phasic syllable-related activity selectively at the first syllable. (**a**) Example of a type-1 unit showing significantly higher phasic activity selectively before the first syllable onset of the song bout. Top panels show spectrograms and amplitude oscillograms of each syllable. Bottom panels show raster plots and spike rate histograms aligned to each syllable. From left to right, each column shows the raster plot and spike rate histogram of the same neuron aligned to the first, second, third, fourth, fifth, and final syllable. Gray shadings indicate the range from − 50 to + 50 ms of each syllable. Data is from the same single unit as in Fig. 1a. (**b**) Example of a type-2 unit showing sustained firing activity. Data is from the same single unit as in Fig. 1b. (**c**) Example of a type-3 unit showing significantly lower phasic activity selectively at the first syllable onset. Data is from the same single unit as in Fig. 1c.

**Figure 4:**
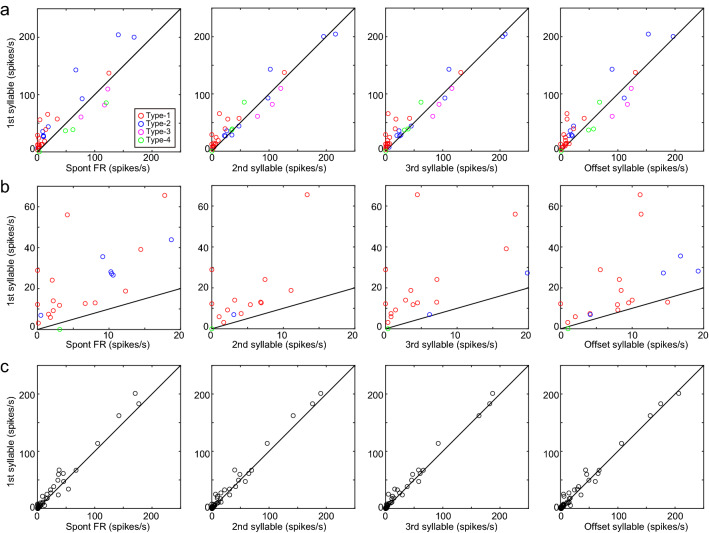
Comparison of syllable-related activity. (**a–c**) From left to right: mean firing rate at the first syllable is plotted against the spontaneous firing rate, mean firing rate at the second syllable, mean firing rate of the third syllable, and mean firing rate at the final syllable. (**a)** Data from singing-related units is shown. (**b**) The low firing rate portion (0–20 Hz) of the same data as in (**a**) are shown for clarity. (**c**). Data from non-singing related units is shown.

**Figure 5:**
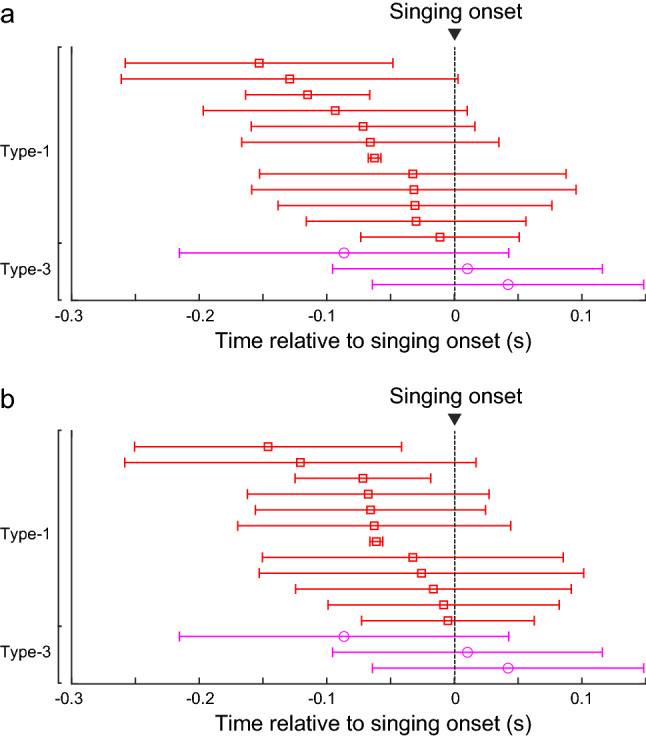
Timing of activity changes for type-1 and type-3 units. (**a**) Onset time of activity change for individual type-1 (red) and type-3 (magenta) units is shown. Red squares and magenta circles represent mean (± SD) time of activity change relative to the song onset. (**b**) Timing of firing rate peaks (type-1, red) or troughs (type-3, magenta) relative to the song onset is shown.

Juvenile zebra finches produce highly variable songs, and inter-syllable intervals vary from rendition to rendition. To examine whether the activity changes seen during singing occurred specifically at the first syllable, we compared the activity relative to each song syllable within the song bout. For each single unit, PETH were calculated on the basis of the alignment to the first, second, third, fourth, fifth, or final syllable of the song bout (Fig. 3). Remarkably, we found that type-1 units exhibited a phasic increase in the firing rate selectively at the first syllable, which was significantly higher than the firing rates at the rest of syllables within the song bout (Fig. 3a, Bonferroni correction for multiple comparison test, p < 0.05, n = 17). Likewise, type-3 units exhibited a phasic decrease in the firing rate selectively at the first syllable (Fig. 3c, p < 0.05, n = 3). On the other hand, type-2 (Fig. 3b) and type-4 units did not show selective changes in activity at any specific syllable. Although we found selective activity changes at the first syllable in type-1 and type-3 units, there were no single units showing selective changes in activity at any other syllable within the song bout (Fig. 4). These analyses showed that type-1 and type-3 units exhibited activity changes specifically at the initiation of song. We further examined the exact time when the activity changed prior to song onset (Fig. 5). For each single unit, we calculated the timing of the activity change for each trial (detection threshold, mean firing rate ± 2 SD), the timing of the firing rate peak (type-1) or trough (type-3), and the duration of the activity change. The changes in activity began 10–150 ms before song onset in type-1 units. In type-3 units, the changes began anywhere from 100 ms before to 40 ms after song onset (Fig. 5a, type-1 units, mean ± SD, − 68.9 ± 45.2 ms, range: − 153.0 to − 11.2 ms relative to song onset, n = 12, type-3 units, − 11.3 ± 67.0 ms, range: − 86.4 to 42.2 ms, n = 3), and quickly reached the firing peak or trough (Fig. 5b, type-1, mean ± SD, − 56.9 ± 43.4 ms, range: − 146.0 to − 5.0 ms relative to song onset, type-3, − 11.3 ± 67.0 ms, range: − 86.4 to 42.2 ms). The duration of the change in activity was about 100 ms (type-1, mean ± SD, 100 ± 48 ms, range: 60 to 170 ms, type-3, 70 ± 17 ms, range: 50 to 80 ms). As a population, most type-1 and type-3 units exhibited a change in activity prior to song initiation (type-1, 12/12 units, type-3, 1/3 units). We also examined whether these single units showed an increase in activity several hundred milliseconds before song initiation, similar to the preparatory activity seen in song nuclei^21^. We found no such gradual activity change (detection threshold, mean firing rate ± 2 SD). These results indicate that the VTA and SNc in juvenile zebra finches contain neurons related to the initiation of song vocalization.

**Figure 6:**
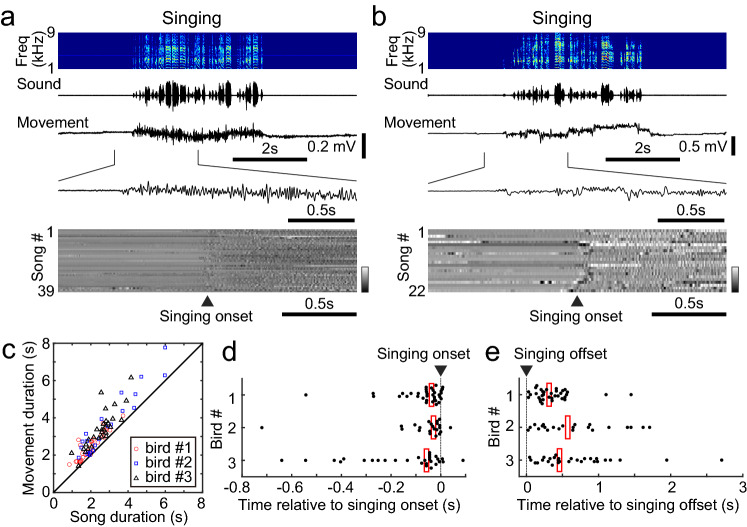
Relationship between song and movement. (**a**) Example of head movement during singing. Spectrogram, amplitude oscillogram of song, and raw movement signal measured using an accelerometer are shown from top to bottom. Gray scale map indicates movement signals during singing. Data is from a 75-day old bird (bird #1). Note that head movements continue until the end of singing. (**b**) Another example of head movement measured from a 58-day old bird (bird #2). (**c**) Comparison between duration of song and movement for 3 birds. Each symbol represents data from a single song epoch. (**d**) Head movement precedes singing onset. Time of movement onset relative to the song onset are plotted for 3 birds. Each dot represents a single song epoch. Red bars indicate median time. (**e**) Head movement ceased after the song ended. Time of movement offset relative to the song offset for 3 birds are plotted in the same manner as (**d**).

Since singing is accompanied by movement in zebra finches, the singing-related activity described above may be also related to the movement of the birds. In fact, previous studies show that some VTA neurons in adult zebra finches code performance error signals while others exhibit movement-related activity as well as syllable-locked activity^32,39^. To elucidate the relationship between singing and movement in juvenile zebra finches, an accelerometer was attached to the bird’s head and movement-related signal was measured during singing (Fig. 6). We observed that continuous head movement occurred during singing in juvenile birds (Fig. 6a,b), and that movement duration was consistently longer than song duration (Fig. 6c, Wilcoxon signed-rank test, p < 0.05, n = 3 birds). Furthermore, when we calculated the time difference between the onsets of song and movement, we found that head movement preceded singing by less than about 100 ms (Fig. 6d, movement onset time relative to song onset, mean ± SD, − 41.4 ± 14.6 ms, n = 3 birds). We also found that movement ceased about several hundred milliseconds after the song ended (Fig. 6e, movement offset time relative to song offset, mean ± SD, 449.1 ± 129.9 ms, n = 3 birds). As mentioned earlier, type-1 units started to change their activity 10–150 ms before the onset of singing (Fig. 5a), and such change in activity sometimes occurred after the head movements were executed. Thus, the phasic activity of some type-1 units seems to be involved in the initiation of song, rather than the initiation of the head movements. However, since we did not quantify detailed body movements other than head movements, we cannot completely rule out the possibility that neural activity is correlated with other non-head movements associated with song initiation. Overall, these results suggest that some of the phasic activity occurring in the VTA/SNc at the initiation of song represents the start signal for song vocalizations, while other examples of phasic activity may represent start signals for movements that accompany singing.

## Discussion

By recording neural activity in singing juvenile male zebra finches, we found that VTA/SNc single units exhibited singing-related activity, and that phasic activity was tightly time-locked to the initiation of song. Some VTA/SNc units exhibited a transient increase or decrease in activity specifically at the initiation of the song bout (Fig. 1, type-1 and type-3 units), while others showed sustained activity during singing (type-2 and type-4 units). These results support the notion that the phasic activity of VTA/SNc neurons represents a start signal that triggers song vocalization in juvenile zebra finches.

Previous studies have demonstrated that neurons in the song system nuclei exhibit preparatory activity prior to the onset of song vocalization. In the basal ganglia circuitry nuclei Area X and LMAN, neural activity gradually increases several hundred milliseconds before the onset of an undirected song bout^22,24,25,40^. Similarly, neurons in the premotor nucleus HVC change their activity several hundred milliseconds before song bout onset^20^. Much longer timescale changes have been demonstrated in RA-projecting HVC neurons and the downstream motor cortical neurons in RA^21^. These preparatory activities in the song system nuclei are thought to play important roles in impending song performance. In contrast, VTA/SNc activity at the onset of the song bout was relatively transient (Fig. 1, type-1 and type-3 units) compared to preparatory activity in the song system nuclei, which can last several hundred milliseconds to a few seconds. We did not find such preparatory activity in the VTA/SNc. Thus, VTA/SNc in juvenile may provide a start signal rather than a preparatory function for song initiation.

The song system nuclei, such as HVC, RA, and Area X, receive dopaminergic inputs from VTA/SNc and PAG^26–28,30^, and are abundant in various types of dopamine receptors^31^. An important question is whether the neural signals propagating from the VTA/SNc can reach the song nuclei and function as song initiation signals. Previous studies have shown that the time delay between the onset of singing-related activity in HVC/RA and song production is about 40–50 ms^41,42^. On the other hand, the time delay between the projection from Area X to RA (via DLM/LMAN) and the generation of song can be roughly estimated as 80–100 ms at most. This is calculated from the latency of LMAN stimulation effects on a song syllable (50 ms at most)^43–45^ and the latency to thalamic DLM spike from the preceding spike decelerations of Area X pallidal output neurons (about 20 ms)^46,47^. The phasic activity in the VTA/SNc that we found in this study arose 10–150 ms before singing onset in type-1 units. In type-3 units, activity occurred anywhere from 100 ms before to 40 ms after song onset (Fig. 5). In contrast to preparatory activity in the song system nuclei, which gradually increases several hundred milliseconds to a few seconds before singing, it is possible that the phasic activity in the VTA/SNc may serve as a final trigger for song vocalization. Such transient dopaminergic inputs from VTA/SNc to the song system nuclei may enhance the excitability of neurons related to song preparation. In addition, both neural activity in Area X and vocal learning are modulated by manipulations of dopaminergic inputs^33–35,48–50^. Thus far, however, the role of dopamine in song initiation has been overlooked, except for a previous report describing pre-singing VTA activity in adult zebra finches^51^. Along with its previously established role in vocal learning and social modulation of vocal performance^16^, our findings indicate a possible novel function of the songbird VTA/SNc dopaminergic system in the initiation of song. Further studies will be needed to determine whether temporally precise transient dopaminergic signals driven by electrical/optogenetic stimulation of VTA/SNc neurons may initiate song in concert with preparatory activity in the song system nuclei.

In adult birds, dopamine release in the basal ganglia nucleus Area X is known to be greater during courtship singing to a female bird (directed song) and less when singing alone (undirected song)^52^. Depending on the social context, both immediate-early gene expression and singing-related activity of the basal ganglia network, as well as the VTA/SNc, differ^25,40,51,53–55^. In this study, we analyzed activity in the VTA/SNc of juvenile birds while they were practicing song alone and found vocal initiation-related activity. This vocal initiation-related activity may change depending on the social context and with developmental stage. It would be worth investigating how vocal initiation-related activity in the VTA/SNc differs in various situations and behavioral states, such as vocal practice in different developmental stages, courtship singing to a female bird, and vocal communication with socially motivated calls.

In summary, the present study shows that a subset of VTA/SNc single units in juvenile male zebra finches exhibit phasic activity at the initiation of song. This result underscores the potential importance of the songbird VTA/SNc for vocal initiation, and suggests a functional similarity to the mammalian dopaminergic system involved in the initiation of various voluntary movements.

## Materials and methods

### Animals

All experiments were approved by the animal experimentation committee at the University of Tokyo and performed in accordance with the established guidelines (permission #27–9 and #29–2). All procedures reported in this study were carried out in compliance with the ARRIVE guidelines. Male zebra finches were obtained from our breeding colony (n = 6). Birds were kept on a 14 L:10 D photoperiod, and food and water were available ad libitum. Single unit recordings were performed from freely behaving juvenile birds (49–83 days post hatch). A total of 85 single units were recorded from 6 birds (recording onset: 49–78 days post hatch, mean ± SD: 66.3 ± 9.6 days).

### Surgery and electrophysiological recordings

Single unit activity was recorded extracellularly from VTA/SNc in freely behaving zebra finches. For this study, we used a handmade, manually advanceable microdrive (1 g in weight) attached to four bundles of tetrode and reference wires (nichrome wire, diameter, 12.5 µm, RO800, Sandvic). Under isoflurane anesthesia (1–1.3%), the microdrive was chronically implanted with a stereotaxic apparatus (Narishige). Before implanting the tetrode, each electrode was plated with gold plating solution (Neuralynx). The impedance of the electrode was 300–500 kΩ. The following stereotaxic coordinates were used; anterior: 0.8–1.0 mm, lateral: 0.5–0.8 mm, depth: 5.6–6.8 mm from the bifurcation of the sagittal sinus, head angle: 28 degrees. After the surgery, birds were kept singly in a recording chamber (30 × 20 × 25 cm), which was placed in a sound attenuation box (50 × 40 × 40 cm). Multiple single-unit activity was recorded while the bird was singing spontaneously. Extracellular neuronal signals were amplified (10,000-fold), band-pass filtered (0.5–9 kHz), and digitized (40 kHz) with a Plexon MAP system. The acoustic signal in the sound attenuation box was simultaneously recorded with a microphone (model C417, AKG) placed inside the sound attenuation box. The acoustic signal was amplified (DMP3, M-AUDIO) and digitized at 20 kHz. The behavior of the bird was monitored with a video camera (CinePlex, Plexon). To measure the bird’s head movement, an accelerometer was mounted on the bird’s head, and the accelerometer signal was recorded at 20 kHz (3 birds). Neural, sound, and accelerometer signals digitized with a Plexon MAP system were stored on a PC.

### Analysis of electrophysiological data and movement

Spike signals were sorted offline into single units (Offline sorter, Plexon), and well-isolated single units were further analyzed with MATLAB (MathWorks). To characterize spike shape for each single unit, first a mean waveform was calculated based on 20 randomly chosen spike waveforms. Then, the spike width was calculated as the half-width of the first negative deflection of the mean waveform. To visualize and compare the change in firing between single units with different firing rates at the onset and offset of song, we displayed the firing rate in z-scores (Fig. 1e). The z-score firing rate was computed so that the mean of the firing rate in the two-second period at the onset/offset of the song was centered to be 0 and the standard deviation was scaled to be 1 (MATLAB function zscore). To determine whether single unit activity was related to singing, the number of spikes before singing (pre-singing period) and during singing were compared. To calculate these values, the onset/offset times of the song bout and the onset times of each syllable (from 1st to 5th syllables in each song bout) were manually determined by visual inspection of the spectrogram and oscillogram of the acoustic signal. The pre-singing period was determined as the period 400–500 ms prior to each song bout onset. For each song, spike numbers in each 100-ms pre-singing period were calculated. To compare the spike numbers for each syllable during singing, each syllable was divided into windows of 100 ms and spike numbers in each 100 ms window during the first, second, third, fourth, fifth, or final syllable of the song bout were calculated, respectively (Fig. 3). For each single unit, spike numbers in the pre-singing period and spike numbers in each syllable timing (100 ms window) during singing were compared using Friedman test, followed by Bonferroni corrected multiple comparison test. The significance level was set at 0.05. If a significant difference was found in the Friedman test, the single unit was considered singing-related. Furthermore, if the Bonferroni corrected multiple comparison test showed a significant increase in firing rate only around the first syllable, the unit was classified as type-1. If there was an increase in firing rate not only around the first syllable, but also in the second and subsequent syllables, the unit was classified as type-2. If there was a significant decrease in firing rate only around the first syllable, the unit was classified as type-3. If there was a decrease in firing rate not only around the first syllable but also in the second and subsequent syllables, the unit was classified as type-4.

To determine the exact time before song onset when activity change occurred, the time points at which the mean firing rate exceeded a threshold was detected in each trial (detection threshold = mean ± 2 SD, bin size = 10 ms, range of examination: − 0.25 to 0.25 s relative to the song onset). In order to detect changes in activity reliably in each trial, units with extremely low firing rates (less than 5 spikes in each trial) were excluded from this analysis. In addition, the timing of the peak (type-1) or trough (type-3) of firing was detected for each trial and the duration for which the firing rate exceeded the threshold was measured. In addition to detecting these transient changes in activity around the onset of singing, the occurrence of gradual activity changes over a longer period of time, such as preparatory activity before the start of singing, was examined (detection threshold = mean ± 2 SD, bin size = 10 ms, range of examination: − 2 to 2 s relative to the song onset). Since vocalization-related units were recorded within a dense population of tyrosine hydroxylase (TH)-positive cells, which indicates the VTA/SNc complex (Fig. 2a,b), these data sets were reported as VTA/SNc single units. To examine the relationship between movement and timing of song, the onset and offset of movements were manually determined by visual inspection of the movement signal from the accelerometer. For each bird, the durations of song and movement were compared with a Wilcoxon signed-rank test. The significance level was set at 0.05.

### Anatomical verification of recording sites

After completion of electrophysiological recordings, small electrical lesions were made through the electrodes (20 µA for 20 s, Stimulus Isolator A365, WPI). Birds were deeply anesthetized with an overdose of pentobarbital sodium (Somnopentyl, Kyoritsu Seiyaku) and perfused with 4% paraformaldehyde (PFA). Brains were dissected out, and post-fixed overnight in 4% PFA followed by 30% sucrose in phosphate-buffered saline. Sagittal brains sections (40 µm in thickness) were made with a freezing microtome (ROM-380, Yamato Kohki Industrial), and stained with an antibody against tyrosine hydroxylase (TH, MAB318, Merck Millipore) to visualize dopaminergic neurons in VTA and SNc. The electrode track was verified under a microscope.

## Data Availability

Datasets generated for this study are available from the corresponding author upon reasonable request.
